# Integrative diagnosis of primary cutaneous large B-cell lymphomas supports the relevance of cell of origin profiling

**DOI:** 10.1371/journal.pone.0266978

**Published:** 2022-04-22

**Authors:** Audrey Gros, Sarah Menguy, Victor Bobée, Océane Ducharme, Isabelle Cirilo Cassaigne, Béatrice Vergier, Marie Parrens, Marie Beylot-Barry, Anne Pham-Ledard, Philippe Ruminy, Fabrice Jardin, Jean-Philippe Merlio

**Affiliations:** 1 Tumor Biology and Tumor Bank Department, University Hospital of Bordeaux, Bordeaux, France; 2 INSERM U1213 BRIC, Team 5, University of Bordeaux, Bordeaux, France; 3 Pathology Department, University Hospital of Bordeaux, Bordeaux, France; 4 Hematology Department, University Hospital of Rouen, Rouen, France; 5 Dermatology Department, University Hospital of Bordeaux, Bordeaux, France; 6 INSERM U1245, Centre Henri Becquerel, University of Normandie, Rouen, France; CNR, ITALY

## Abstract

Primary cutaneous large B-cell lymphomas (PCLBCL) represent a diagnostic challenge because they are classified as PCLBCL, leg type (PCLBCL, LT) or primary cutaneous follicle centre lymphoma, large cell (PCFCL, LC), which differ by prognosis and therapeutic requirement. Unclassified cases with discordant clinical presentations, morphologies, and immunophenotypes may be classified into the not otherwise specified (PCLBCL, NOS) category based on ancillary molecular analyses. Cell-of-origin profiling as germinal centre (GC) type or non-GC type by immunohistochemistry is not considered reproducible because of variable CD10 expression. In a series of 55 PCLBCL cases with > 80% large cells, we reported 21 PCFCL, LC cases as GC-type and 27 PCLBCL, LT as non-GC-type; 7 cases were considered PCLBCL, NOS. Here, we demonstrate the accuracy of molecular profiling of PCLBCL as GC or non-GC type using a reverse transcriptase multiplex ligation assay (RT-MLPA). RT-MLPA classified the seven PCLBCL, NOS cases in accordance with their mutational profile. An integrative principal component analysis confirmed the main criteria and the relevance of genomic profiling of PCFCL, LC as GC-derived, and PCLBCL, LT as non-GC-derived. Both the cell-of-origin classification of PCLBCL and the integrative analysis identified two clinically relevant subgroups according to overall survival, which may help to standardize PCLBCL diagnosis and patient management.

## Introduction

Primary cutaneous large B-cell lymphomas (PCLBCL) are characterized by a dense dermal infiltrate with a diffuse and/or nodular pattern composed of > 80% large tumor cells corresponding to centroblasts and/or immunoblasts, and a proliferative index ≥ 40%. It is important to differentiate primary cutaneous follicle centre, large cell (PCFCL, LC) from primary cutaneous large B-cell lymphoma, leg type (PCLBCL, LT), because they differ by prognosis and therapeutic requirements [1–4].

Patients with PCFCL, LC are mostly treated by local approaches such as radiotherapy or, if they have multiple lesions, by rituximab only [5–7]. Despite possible cutaneous relapse, they generally have an indolent course with a 5-year overall survival (OS) rate of > 95% [6]. PCFCL, LC includes a variant with large, cleaved cells in a collagenous stroma that is generally easy to identify. Because all PCFCL subtypes have the same prognosis and are treated similarly, they are in the same category (PCFCL) in the 2016 World Health Organization (WHO) classification of tumors of hematopoietic and lymphoid tissues, and in the 2018 WHO classification of skin tumors [1, 3].

Alternatively, PCLBCL, LT may have an aggressive course, requiring a combination of rituximab and polychemotherapy as first-line therapy [5, 7, 8]. Such treatment improves the specific survival (SS) of patients with PCLBCL, LT but about one-half will experience therapeutic resistance or relapse, leading to a shorter SS [8].

Due to discordance among clinical, morphological, and immunophenotypic features, rare PCLBCL cases cannot be classified into the above two categories. Both the 2018 WHO classification of skin tumors and the 2018 WHO-EORTC classification of primary cutaneous lymphomas recommend the term *not otherwise specified* (PCLBCL, NOS) [2, 4].

A cell-of-origin classification of PCLBCL by genomic profiling of a restricted set of frozen material indicated that PCFCL, LC exhibits a germinal centre (GC) signature, whereas PCLBCL, LT has a non-GC signature [9]. For paraffin-embedded material, the use of Hans’ algorithm is not accepted for PCLBCL, unlike systemic diffuse large B-cell lymphoma (DLBCL) [1, 4]. Indeed, the 2018 WHO classification of skin tumors and 2018 WHO-EORTC guidelines recommend use of morphological criteria (such as confluent sheets of centroblasts and/or immunoblasts) or phenotypic criteria (such as strong expression of BCL2, MUM1, FOXP1, and cytoplasmic IgM and CD10 negativity) for the diagnosis of PCLBCL, LT [2, 4]. A few PCLBCL, LTs may lack this typical immunophenotype, such as those without BCL2 expression [4, 10]. Moreover, CD10-negative PCFCL, LC and CD10-positive PCLBCL, LT have been reported, underscoring the limitations of CD10 assessment in cutaneous B-cell lymphomas [11, 12]. Combining the above criteria with a simplified Hans’ algorithm, we previously evaluated 55 PCLBCL cases classified as PCFCL, LC (GC type; n = 21) or PCLBCL, LT (non-GC type; n = 27); 7 cases were unclassified or NOS prior to molecular analysis [13]. PCLBCL, LT showed highly recurrent mutations of *MYD88* (p.L265P variant), *PIM1*, and *CD79B*, which are involved in the NF-κB and B-cell receptor signaling pathways, similar to primary central nervous system lymphoma (PCNSL) [14, 15].

PCLBCL, NOS diagnostic accuracy may differ according to local or centralized expertise and the availability of molecular analyses [4]. No integrated study has evaluated the concordance of clinical, morphological, phenotypic, and molecular WHO-EORTC criteria in typical and unclassified cases.

Here, we used a reverse transcriptase multiplex ligation assay (RT-MLPA) applicable to fixed specimens to establish the cell of origin as GC or non-GC in 55 PCLBCL cases [13, 16]. The mutational profile was assessed by next-generation sequencing. Through integrative principal component analysis (PCA), we evaluated cell-of-origin subtype and clinical, phenotypic, and genetic criteria for classifying PCLBCL as PCFCL, LC or PCLBCL, LT. The clinical relevance of the cell of origin or integrative classification of PCLBCL was evaluated according to OS.

## Methods

### Patients

The 55 PCLBCL cases showing a dermal infiltrate with a diffuse and/or nodular growth pattern comprised of at least 80% large B-cells were selected from our previous study of 64 cases; 9 cases were excluded because the material was exhausted [13]. All cases were retrieved from the French Study Group of the Cutaneous Lymphoma Database (S1 Table). Diagnosis was validated during multidisciplinary consultation meetings attended by dermatologists and dermatopathologists using the WHO 2017 classification criteria [13]. All patients had primary cutaneous disease, as determined by negative initial staging including computed tomography (CT), and an at least 6-month follow-up without evidence of systemic lymphoma. The cases were reviewed by our team according to the clinical and histopathological WHO-EORTC classification criteria [4]. Briefly, formalin-fixed paraffin-embedded (FFPE) slides were subjected to automated immunolabelling (Bond Max; Leica, Nussloch, Germany) with MUM1 (clone MUM1p; Agilent-Dako, Glostrup, Denmark), CD10 (clone 56C6, Leica), BCL6 (clone PGB6P, Agilent-Dako), and BCL2 (clone 124; Agilent-Dako). The positivity cut-offs for BCL2, BCL6, MUM1, and CD10 were, respectively, ≥ 50%, ≥ 30%, ≥ 30%, and ≥ 30% of cells. GC and non-GC profiles were assessed using the Hans algorithm [13]. Immunostaining for IgM was performed using clone 8H6 (Leica) and scored as positive above a 50% cutoff [17]. Immunostaining for CD21 with clone 2G9 (Leica) was performed to identify follicular dendritic cell networks (FDCNs). The study was performed anonymously after the patients had provided informed consent according to the French bioethics law for non-interventional studies. Formal approval was granted by the Research Ethics Committee of the University Hospital of Bordeaux.

### RT-MLPA

For nodal DLBCL, Bobée *et al*. developed a novel gene expression profiling DLBCL classifier based on RT-MLPA [16]. Through semi-quantitative methods, it evaluates the expression of 21 markers, including the following GC-expressed genes: *ITPKB*, *LMO2*, *MAL3*, *MME*, *MYBL1*, and *NEK* (versus activated B-cell or non-GC expressed genes such as *IRF4*, *FOXP1*, *IGHM*, *TNFRSF13B*, and *LIMD1*). It also measures the expression of *MYC*, *CD5*, *CRBN*, and *CCND1* and of the LMP-1 Epstein-Barr virus (EBV) protein. The technique also detects MYD88L265P variant transcripts but requires ≥ 25% tumor cells, estimated based on CD20 expression. A sample score for the assay is generated via a web interface to differentiate primary mediastinal B-cell lymphoma, activated B-cell-like, GC B-cell-like, and EBV-positive DLBCLs (http://92.222.23.215/RTMLPA). Briefly, RNA was extracted from paraffin sections using the Maxwell Promega RNA Extraction Kit (Promega, Madison, WI, USA), or from frozen material (when available) using the RNeasy Plus Mini kit (Qiagen, Courtaboeuf, France). cDNA was generated by reverse transcription; RT-MLPA probe mix was added, and PCR products were analyzed by fragment analysis. The profiles were determined as GC, non-GC, and non-contributive (NC). The technique was also found reproducible between 5 French laboratories participating to a national trial and quality control on the same samples (data not shown).

### Next-generation sequencing using a lymphopanel targeting 36 genes involved in B-cell lymphomagenesis

DNA was extracted from FFPE (n = 30) and frozen tissues (n = 25). The lymphopanel was designed with Ion AmpliSeq technology (ThermoFisher Scientific, Waltham, MA, USA) and covered 75.08 kb. We focused on 36 genes involved in lymphomagenesis based on literature data and whole-exome sequencing (WES) of relapsed/refractory DLBCL. The genes included *ARID1A*, *B2M*, *BCL2*, *BRAF*, *BTK*, *CARD11*, *CCND3*, *CD58*, *CD79A*, *CD79B*, *CDKN2A*, *CDKN2B*, *CIITA*, *CREBBP*, *CXCR4*, *EP300*, *EZH2*, *FOXO1*, *GNA13*, *ID3*, *IRF4*, *MEF2B*, *MYC*, *MYD88*, *NOTCH1*, *NOTCH2*, *PIM1*, *PLCG2*, *PRDM1*, *SOCS1*, *STAT6*, *TCF3*, *TNFAIP3*, *TNFRSF14*, *TP53*, and *XPO1* [18]. Amplified libraries were sequenced with the Ion 530 Chip kit and data were analyzed with Torrent Suite™ software (version 5.10; ThermoFisher Scientific). Reads were mapped to the human hg19 reference genome. The variant caller detected point mutations with a variant allele frequency (VAF) ≥2% for single nucleotide variations and ≥ 5% for short insertions/deletions. VCF files were annotated by ANNOVAR [19]. The BAM sequence was checked using Alamut Visual Software (SOPHiA Genetics, St. Sulpice, Switzerland).

### Principal component analysis (PCA)

PCA was performed using the R packages FactoMineR and Factoshiny [20]. This tool compresses the information contained within a number *p* of variables into a smaller set of *q* factors. No scaling is applied to rows. Supervised dimension reduction with imputation is used to calculate principal components. The X- and Y-axes show principal components 1 and 2. This tool reduces the dimensionality of datasets and highlights variables that support individual mapping. We first evaluated the 48 typical PCLBCL cases using the following criteria: limb involvement, individual positive immunostaining (CD10, BCL6, MUM1, BCL2, FDCN, CD21), RT-MLPA profile (GC *vs*. non-GC), and individual mutations. We then applied this tool to the seven undetermined or provisional NOS cases.

### Statistical analysis

Quantitative characteristics are described as medians and ranges, and qualitative characteristics as numbers and frequencies. Comparisons of qualitative characteristics between subgroups were performed using Fisher’s exact test. Progression-free survival (PFS) was determined as the time between the date of diagnosis and date of recurrence or death. OS was determined from the date of diagnosis to the date of death of any cause. SS was calculated from diagnosis to disease-related death, where patients whose death was unrelated to lymphoma were considered censored. Kaplan-Meier survival curves were plotted for each prognostic factor and compared using the log-rank test. A two-sided p < 0.05 was considered significant. Statistical analysis was performed using MedCalc software (version 12.4.0; MedCalc Software, Ostend, Belgium).

## Results

### Clinical features and distribution of histological diagnoses

**Fig 1:**
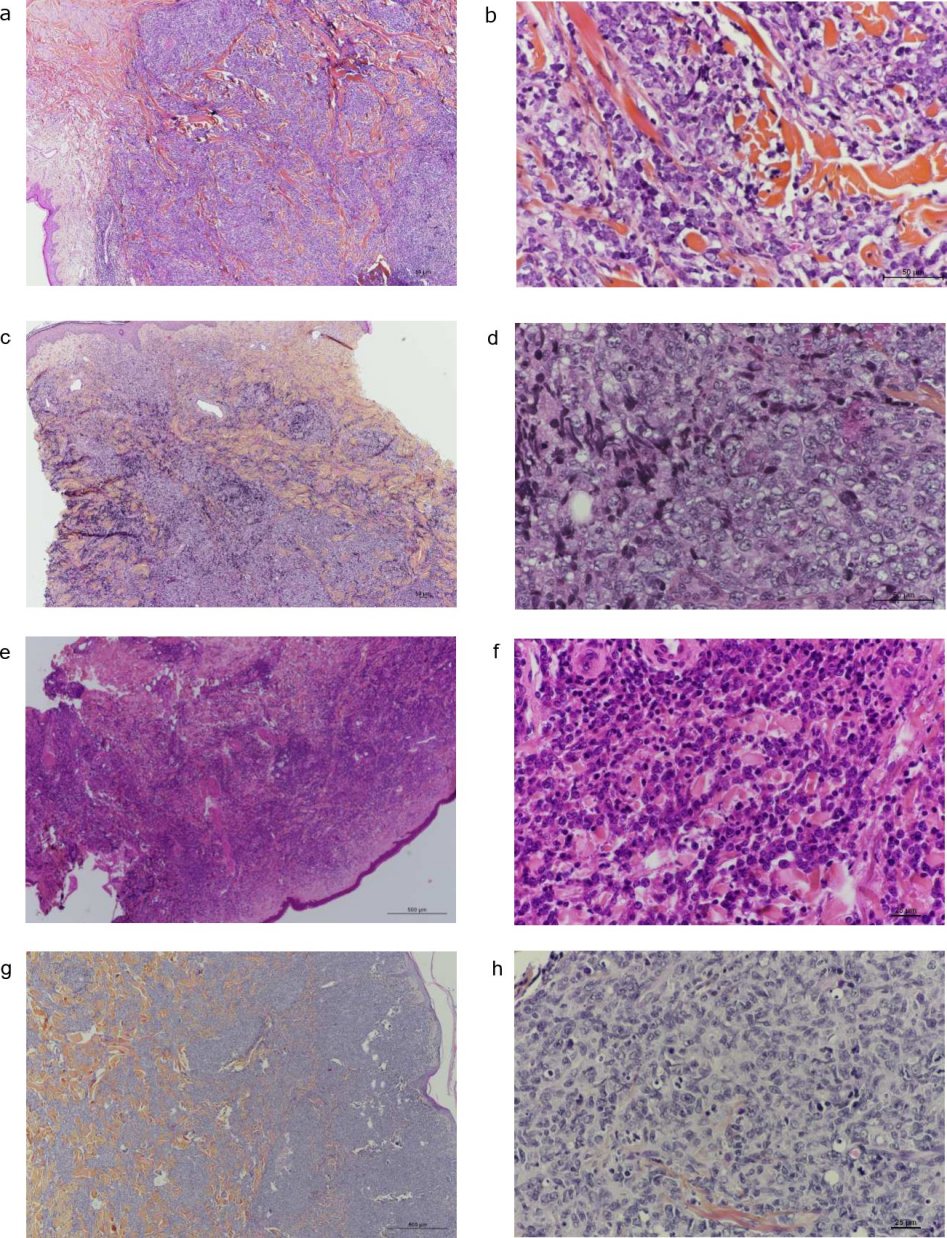
Histopathological aspects of PCLBCLs. a. PCFCL, LC (LC-1): HES x4: nodular and diffuse lymphoid dermal infiltrate. b. PCFCL, LC (LC-1): HES x40: large tumoral cells with cleaved or elongated nuclei. c. PCLBCL, LT (LT-23): HES x4: nodular and diffuse lymphoid dermal infiltrate. d. PCLBCL, LT (LT-23): HES x40: large tumoral cells with a mixture of centroblasts and immunoblasts. e. PCLBCL, LT (LT-2): HES x4: nodular and diffuse lymphoid dermal infiltrate. f. PCLBCL, LT (LT-2): HES x40: large tumoral cells with a predominance of immunoblasts. g. PCLBCL, NOS (NOS-1): HES x4: nodular and diffuse lymphoid dermal infiltrate. h. PCLBCL, NOS (NOS-1): HES x40: large tumoral cells with either centroblastic, immunoblastic or cleaved cytology. HES: Hematein-Eosin-Saffron; PCLBCL, LT: primary cutaneous large B-cell lymphoma, leg type; PCFCL, LC: primary cutaneous follicle centre lymphoma, large cell; PCLBCL, NOS: primary cutaneous large B-cell lymphomas, not otherwise specified.

Using the 2018 WHO-EORTC clinical, morphological, and phenotypic criteria, the 55 PCLBCL cases were classified as follows: 21 PCFCL, LC (Fig 1A and 1B) and 27 PCLBCL, LT (Fig 1C–1F) cases. Seven cases (13%) were unclassified and provisionally assigned as PCLBCL, NOS (Fig 1G and 1H). They had discordant clinical or morphological features or phenotypes prior to molecular analysis (Table 1).

**Table 1:** Clinical presentation, morphology, phenotype, molecular profile and outcome of the 7 primary cutaneous large B-cell lymphomas not otherwise specified cases.

Provisional classification	Age (y), Gender	Site	Clinical profile	Architecture	Cytology	Morphological profile	Phenotype	Hans	Phenotypical profile	Molecular status or integrated analysis
NOS-1	42, M	trunk	**PCFCL**	N	30%IB 50%CB 0%LSC	**PCFCL, LC** because of nodular pattern but sheets of IB	CD10- BCL6^+^ MUM1^+^ BCL2^+^	non-GC	**PCLBCL, LT**	**PCFCL, LC**
NOS-2	88, F	leg	**PCLBCL, LT**	N and D	50%IB 30%CB 20%LSC	**Undetermined** because of pleomorphic cytology	CD10^+^ BCL6^+^ MUM1^+^ BCL2^+^	GC	**Undetermined**	**PCLBCL, LT**
NOS-3	78, F	leg	**PCLBCL, LT**	D	50%IB 30%CB 20%LSC	**Undetermined** because of pleomorphic cytology	CD10^-^ BCL6^+^ MUM1^+^ BCL2^+^	non-GC	**PCLBCL, LT**	**PCFCL, LC**
NOS-4	74, F	neck	**Unusual**	D	60%IB 40%CB 0%LSC	**PCLBCL, LT** because of majority of IB	CD10^–^ BCL6^+^ MUM1^–^ BCL2^–^	GC	**PCFCL**	**PCFCL, LC**
NOS-5	83, F	trunk	**Unusual**	D	80%IB 20%CB 0%LSC	**PCLBCL, LT** because of majority of IB	CD10^–^ BCL6^+^ MUM1^–^ BCL2^+^	GC	**Undetermined**	**PCFCL, LC**
NOS-6	72, F	head	**PCFCL**	N	60%IB 40%CB 0%LSC	**Undetermined** because of nodular pattern with sheets of IB	CD10^–^ BCL6^+^ MUM1^+^ BCL2^+^ FDCN CD21^+^	non-GC	**Undetermined**	**PCLBCL, LT**
NOS-7	81, M	leg	**PCLBCL, LT**	D	20%IB 60%CB 20%LSC	**Undetermined** because of pleomorphic cytology	CD10^–^ BCL6^–^ MUM1^+^ BCL2^+^	non-GC	**PCLBCL, LT**	**PCLBCL, LT**

M: male; F: female; PCFCL, LC: primary cutaneous follicle centre lymphoma, large cell; PCLBCL, LT: primary cutaneous large B-cell lymphoma, leg type; PCLBCL, NOS: primary cutaneous large B-cell lymphoma, not otherwise specified; Unusual clinical presentation relied on site, appearance or onset speed. LSC: large spindle-shaped centrocytes; IB: immunoblasts; CB: centroblasts; D: diffuse; N and D: nodular and diffuse; N: nodular; FDCN: follicular dendritic cell network revealed by CD21 immunostaining; GC: germinal centre; non-GC: non-germinal centre. Molecular status was determined based on RT-MLPA profile and mutational pattern, except for NOS-1 (classified on RT-MLPA only). Integrated analysis was performed by principal component analysis.

The characteristics of the patients are listed in S1 Table. Cytoplasmic IgM was detected both in PCFCL, LC (4 of 21, 19%) and PCLBCL, LT (9 of 20, 45%) cases. A FDCN was detected by immunostaining for CD21 in a subset of PCFCL, LC (10 of 21, 48%) cases and one PCLBCL, NOS case, but was absent in the PCLBCL, LT group.

### Concordance between GC/non-GC status and RT-MLPA

The Hans and the Hans-modified algorithms classified the 21 PCFCL, LC cases as GC type and the 27 PCLBCL, LT cases as non-GC type [13].

By independent RT-MLPA analysis, all PCFCL, LC cases (n = 21) were classified as GC type and 25 of 27 PCLBCL, LT as non-GC type. Two PCLBCL, LT cases were not characterized because of the presence of < 20% of tumor cells in the remaining material. The immunohistochemical and RT-MLPA profiling techniques agreed regarding the categorization of typical PCFCL, LC cases as GC type and typical PCLBCL, LT cases as non-GC type (Tables 2 and S2). Among the seven provisional NOS cases, profiling by the two techniques was concordant in four: two with GC and two with non-GC status. In three cases, RT-MLPA indicated a status opposite that of the Hans profile (Tables 2 and S2). LMP-1 EBV protein was not detected by RT-MLPA in any case.

**Table 2:** Determination of cell of origin status (Germinal centre GC or Non-GC) according to the Hans’ algorithm, RT-MLPA assay and mutational analysis of 55 primary cutaneous large B-cell lymphomas.

		WHO-EORTC criteria		
		PCFCL, LC (n = 21)	PCLBCL, LT (n = 27)	PCLBCL, NOS (n = 7)
**Cell of Origin**	**Hans:**	**GC = 100%** (n = 21)	**Non-GC = 100%** (n = 27)	**GC = 43%** (n = 3)
				**Non-GC = 57%** (n = 4)
	**RT-MLPA:**	**GC = 100%** (n = 21)	**Non-GC = 93%** (n = 25)	**GC = 57%** (n = 4)
			**ND: 7%** (n = 2)	**Non-GC = 43%** (n = 3)
	**Concordance:**	*100%*	*93%*	*57%*
**Mutational pattern**		**PCFCL, LC** = 33% (n = 7)	**PCLBCL, LT** = 85% (n = 23)	**PCFCL, LC** = 43% (n = 3)
		**Non-specific** = 48% (n = 10)	**Non-specific** = 15% (n = 4)	**PCLBCL, LT** = 43% (n = 3)
		**No mutation** = 19% (n = 4)		**No mutation** = 14% (n = 1)
**Molecular Classification**		**PCFCL, LC** = 100% (n = 21)	**PCLBCL, LT** = 100% (n = 27)	**PCFCL, LC** = 57% (n = 4)
				**PCLBCL, LT** = 43% (n = 3)
**RT-MLPA and mutational pattern concordance**		*100%*	*100%*	*100%*

PCFCL, LC: primary cutaneous follicle centre lymphoma, large cell; PCLBCL, LT: primary cutaneous large B-cell lymphoma, leg type; PCLBCL, NOS: primary cutaneous large B-cell lymphoma, not otherwise specified; GC: germinal centre. NC: non-contributive RT-MLPA assay. The mutational pattern of PCFCL, LC was defined by *TNFRSF14* mutations and that of PCLBCL, LT by *MYD88* mutations or co-occurrence of *CD79B* and *PIM1* mutations. Cases without these mutations were classified as non-specific mutational pattern. Molecular classification was based on the RT-MLPA profile and mutational pattern, or on the RT-MLPA profile only when no specific mutation was detected.

### Lymphopanel analysis by next-generation sequencing (NGS)

Lymphopanel analysis of 50 patients (17 PCFCL, LC, 27 PCLBCL, LT and 6 PCLBCL, NOS) identified 274 mutations in 28 of 36 genes and 50 truncating mutations (frameshift, nonsense, or splice mutations).

PCFCL, LC, was enriched for mutations in immunity pathways such as *TNFRSF14* and *B2M* and epigenetic regulators like *CREBBP* and *EP300* (S1 Fig). *TNFRSF14* mutations were associated with a diagnosis of PCFCL, LC (Fisher’s exact test p = 0.002). No mutations in genes encoding members of the BCR subunit (*CD79A/B*) were detected in this group. Some PCFCL, LC cases (n = 2) displayed a *PIM1* mutation corresponding to a single nucleotide variant, according to a comparison to the multiple *PIM1* mutations generated by ongoing aberrant hypermutation in the PCLBCL, LT and non-GC groups (S1 Fig). No mutation supporting the diagnosis of PCFCL, LC was detected in 14 of 21 of cases (10 cases with non-specific mutations and 4 cases without mutations).

In the PCLBCL, LT group, mutations of *CD79B* involving the BCR signaling pathway and enrichment of mutations of genes in the NF-κB signaling pathway were observed (S1 Fig). Mutations in *MYD88*, *CD79B* and *PIM1* were each associated with a diagnosis of PCLBCL, LT (Fisher’s exact test p < 0.0001). *MYD88* mutation was specific to PCLBCL, LT and had a high prevalence (~80%) (n = 22 of 27) at a VAF of 9–58%. In 2 of these 22 cases, a non-L265P hotspot mutation (at amino acid 217 or 219) was detected. For one patient with *MYD88* wild-type status, the occurrence of multiple mutations in *PIM1* and *CD79B* enabled discrimination of PCLBCL, LT from PCFCL, LC. Finally, 23 of 27 PCLBCL, LT cases exhibited a specific mutational profile and the remaining 4 had non-specific mutations (Tables 2 and S2 and S1 Fig). Comparing the RT-MPLA and NGS data, there was general agreement in most PCFCL, LC and PCLBCL, LT cases (Table 2).

### Integrative PCA classification

PCA was performed to analyze the contributions of limb involvement, individual phenotypic markers, RT-MLPA profile, and mutations to the diagnosis of PCFCL, LC and PCLBCL, LT.

**Fig 2:**
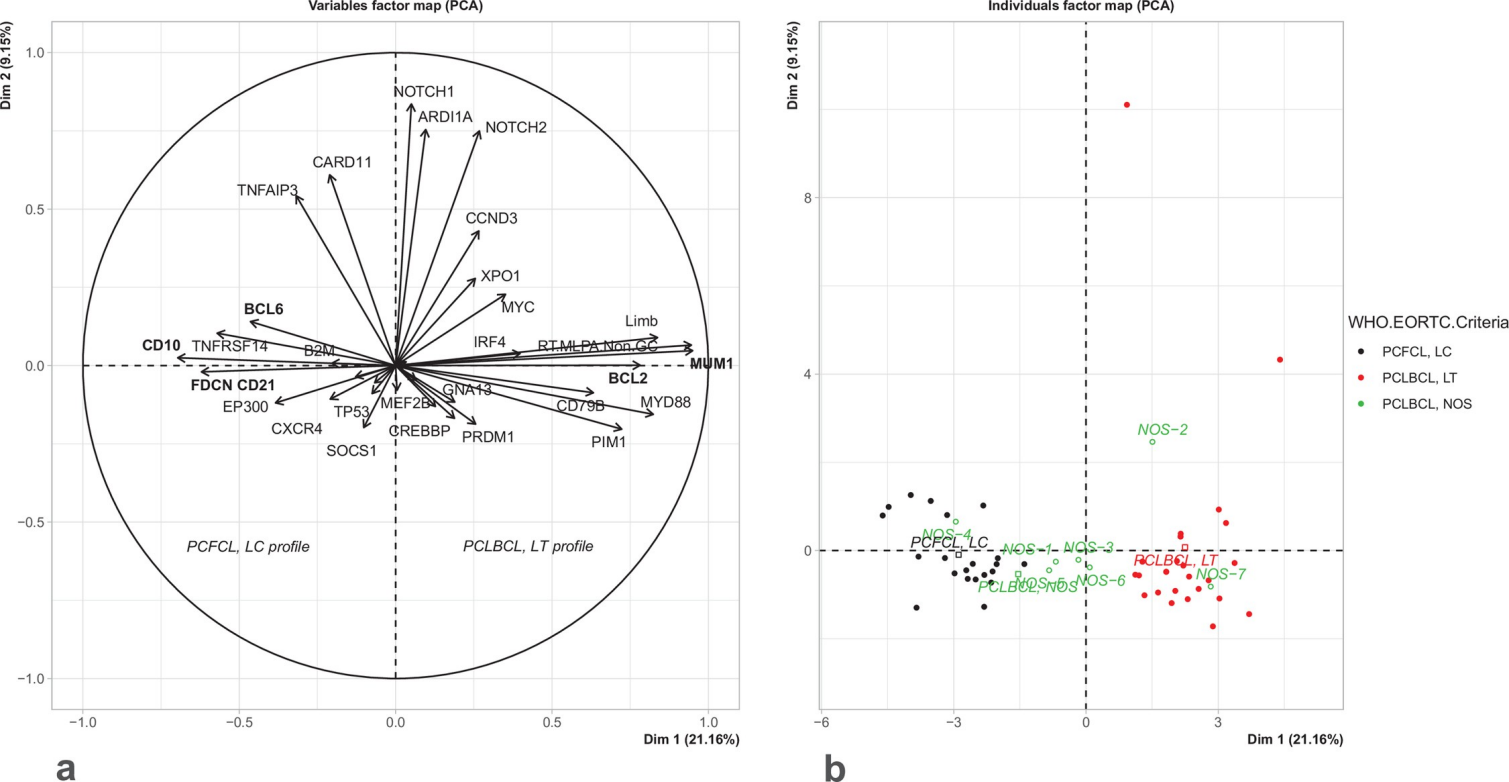
Principal component analysis of the expression profiling by RT-MLPA and mutational analysis. a. Variables factor map, b. Individuals factor map. PCLBCL, LT: primary cutaneous large B-cell lymphoma, leg type; PCFCL, LC: primary cutaneous follicle centre lymphoma, large cell; PCLBCL, NOS: primary cutaneous large B-cell lymphomas, not otherwise specified. Positive immunostaining are in bold by contrast to mutated genes. Principal component 1 and 2 explain 21.16% and 9.15% of the total variance, respectively.

The variable factor map showed that limb involvement and BCL2 and MUM1 expression were strongly associated with a PCLBCL, LT diagnosis. Conversely, CD10 and BCL6 expression and the presence of a CD21+ FDCN were associated with a PCFCL, LC diagnosis. A non-GC RT-MPLA profile was strongly associated with a PCLBCL, LT diagnosis. Genetic mutations were highlighted by PCA in PCFCL, LC (*TNFRSF14*) and PCLBCL, LT (*CD79B*, *MYD88*, or *PIM1)* (Fig 2A).

We next applied our integrative PCA to the seven PCLBCL, NOS cases (Table 1 and Fig 2). A PCA map classified them as follows: PCLBCL, NOS-1, 3, 4 and 5 as PCFCL, LC and PCLBCL, NOS-2, 6 and 7 as PCLBCL, LT (Fig 2B). Three samples (NOS-2, 6, and 7) exhibited a PCLBCL, LT mutational profile with a *MYD88* mutation, with or without *CD79B* or *PIM1* mutation, in accordance with a non-GC profile (S2 Table). Interestingly, NOS-2 occurred on the leg but had a pleomorphic morphology together with strong CD10 expression (S1 Table). In three other cases (NOS-3, 4 and 5), the mutational profile and PCA supported a PCFCL, LC diagnosis with at least a *TNFRSF14* mutation, in accordance with the GC RT-MLPA profile (S2 Table and Fig 2B). Interestingly, NOS-3 could be classified as PCLBCL, LT based on its clinical presentation and phenotype, but presented with a GC profile; moreover, the mutational status supported a PCFCL, LC diagnosis (Table 2). Finally, the NOS-1 case lacked any mutation despite a high content of tumor cells (60%); this would be an unusual feature for PCLBCL, LT (Figs 1 and S1). This case was classified as PCFCL, LC according to the RT-MLPA profile and PCA; this is in accordance with the clinical presentation, but not with a PCLBCL, LT phenotype (Table 1).

### Groups categorized according to molecular features showed different prognoses

**Fig 3:**
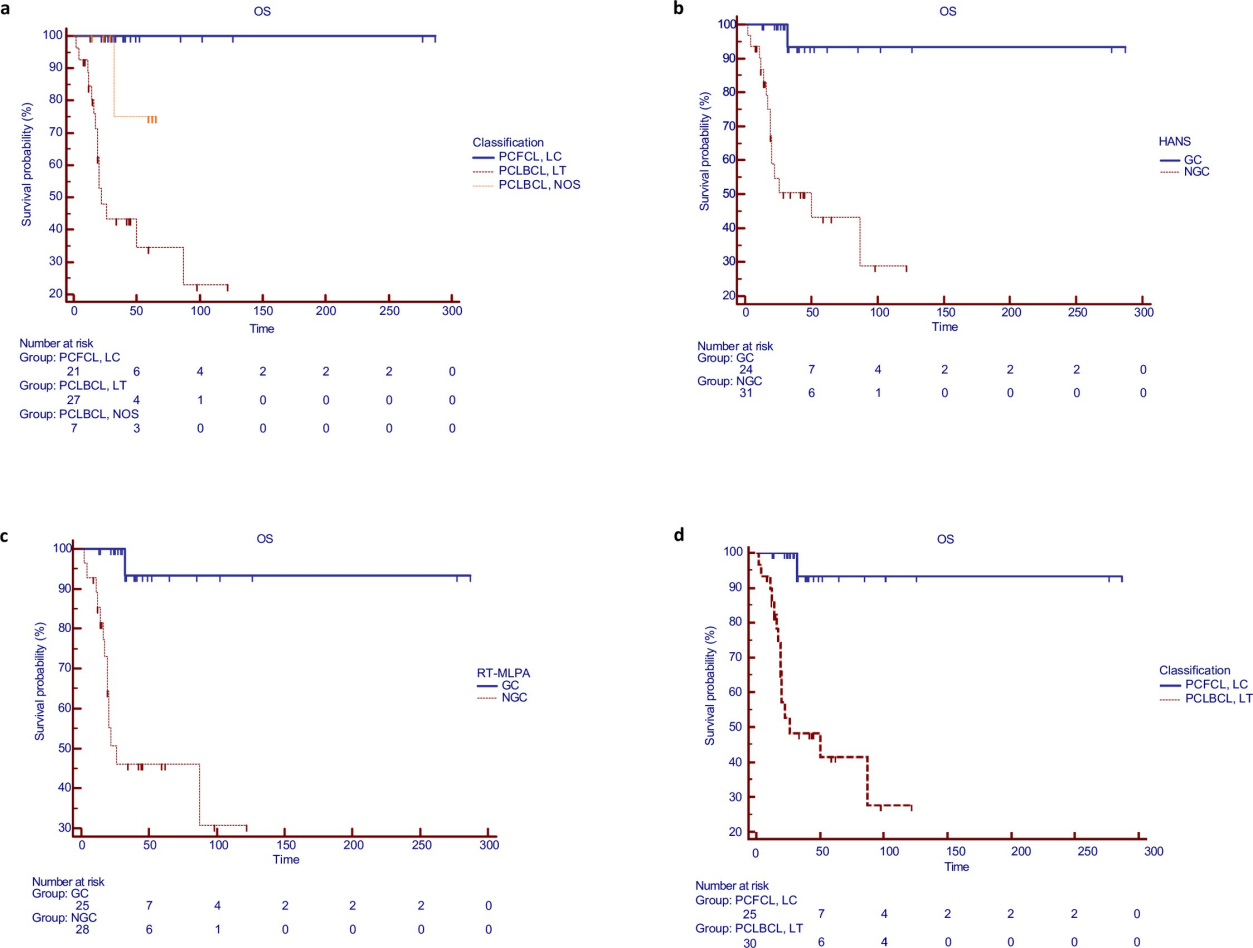
Survival analysis according to the histopathological diagnosis or to the molecular profiling. a. Overall survival (OS) according to the 3 categories of PCLBCL classified according to the 2018 WHO-EORTC criteria. b. OS according to GC and non-GC subtypes defined by Hans’algorithm. c. OS according to GC and non-GC subtypes defined by RT-MLPA assay. d. OS according to the classification by integrative molecular analysis. PCLBCL, LT: primary cutaneous large B-cell lymphoma, leg type; PCFCL, LC: primary cutaneous follicle centre lymphoma, large cell; PCLBCL, NOS: primary cutaneous large B-cell lymphomas, not otherwise specified; OS: overall survival; GC: germinal centre; non-GC: non germinal centre.

Although no difference was seen in PFS, OS and SS (data not shown) were poorer in patients with PCLBCL, LT than PCFCL, LC (p = 0.0001 and 0.0014, respectively) (Fig 3A). The provisional PCLBCL, NOS cases had intermediate survival. Non-GC cases had poorer OS than GC cases according to Hans’ algorithm (p = 0.0002) (Fig 3B) or RT-MLPA (p = 0.0001) (Fig 3C). After reclassification of PCLBCL, NOS into PCFCL, LC or PCLBCL, LT according to PCA, patients with PCLBCL, LT had a poorer OS than those with PCFCL, LC (p = 0.0001) (Fig 3D).

## Discussion

We determined the cell of origin and molecular profile of 55 PCLBCL cases initially characterized by their clinical presentation, morphology, and immunophenotype as PCFCL, LC (n = 21), PCLBCL, LT (n = 27), or provisional NOS (n = 7). The data confirm our findings from Hans’ algorithm analysis of typical cases, and suggest that PCFCL, LC is of GC origin whereas PCLBCL, LT is of non-GC origin [13]. The cell-of-origin profile was concordant with the mutational analysis results.

Key clinical and histopathological features have been used to discriminate PCLBCL, LT lymphoma from other cutaneous B-cell lymphomas [4, 10]. Such disease most frequently involves the limbs, occurs in the elderly with a female predominance, and has a poor prognosis (5-year OS of ~54%), in contrast with the indolent course of patients with other cutaneous B-cell lymphomas including PCFCL [6, 8]. By contrast, PCFCL, LC have never been considered as primary cutaneous GC DLBCL, or as a separate entity; instead, they are grouped with common PCFCL according to their good prognosis [3, 4]. Unlike the WHO recommendations for DLBCL [21, 22], the GC and non-GC Hans’ algorithm have not been accepted for PCLBCL classification [4, 10]. The diagnosis of PCLBCL, LT mainly relies on clinical features, the presence of confluent sheets of centroblasts and/or immunoblasts, and BCL2, MYC, IgM, and FOXP1 expression, irrespective of CD10 expression [4]. Indeed, CD10-positive PCLBCL, LT and PCFCL, LC cases lacking CD10 expression have been reported [11]. In addition, PCFCL exhibits diminished CD10 expression compared with other follicular lymphoma subtypes [12, 23]. Using the Hans algorithm, our Dutch colleagues reported a GC profile in all PCFCL, LC cases (n = 15) but also in 25% of PCLBCL, LT cases (13 of 51), which displayed a CD10+ (n = 7) or BCL6+ MUM1- phenotype (n = 6) [24]. In our series, IgM expression was not discriminant, being detected in 20% of PCFCL, LC cases and 45% of PCLBCL, LT cases; this differs from the 94% rate of IgM expression in PCLBCL, LT cases in the Dutch series [24]. The low rate of IgM-positive PCLBCL, LT cases may result from a sensitivity defect. However, we detected IgM expression in 20% of PCFCL, LC cases, indicating that non-switched IgM-positive B-cells may give rise to cutaneous lymphomas other than PCLBC, LT, including marginal zone lymphomas and PCFCL, LC [25]. The existence of a third subgroup, PCLBCL, NOS, underscores the difficulty of PCLBCL diagnosis, as illustrated by the seven provisional NOS cases classified after expert panel review prior to molecular analyses. Because NOS diagnosis may differ according to the combination of clinical, morphological, phenotypic, and molecular criteria used, we conducted PCA to identify key criteria, including individual mutations, permitting reclassification of these cases.

Here, genomic profiling by RT-MLPA was applied to 53 FFPE specimens of 55 PCLBCL and supported a cell-of-origin classification [13]. A GC profile was found in all 21 PCFCL, LC cases and a non-GC profile in 25 of 27 PCLBCL, LT cases; there were only 2 cases classified as NC, the left-over sample of which contained < 20% large B-cells. The RT-MLPA profile contributed to reclassification of the seven PCLBCL, provisional NOS cases.

Using the NanoString Lymph2Cx gene expression profiling technique, the Dutch group mentioned above recently reported a GC signature in all PCFCL, LC cases (n = 15) [24]. However the 51 PCLBCL, LT cases displayed a GC (39%), unclassified (43%), or NGC/activated B-cell (18%) profile [24]. Preanalytical factors such as RNA degradation from archival specimens may account for the rate of unclassified cases of the Dutch series conversely to ours (4%) because we used RNA extracted from frozen specimens when RNA extracted from fixed material was not informative. The RT-MLPA technique yielded concordant data with the Affymetrix technique for nodal DLBCL [16].

Indeed, our lymphopanel analysis showed that the mutational profile of PCFCL, LC and PCLBCL, LT cases was in agreement with their GC or NGC profile. Our PCLBCL, LT cases exhibited a mutational profile resembling that of PCNSL, with a high rate (80%) of mutations in the TLR MYD88 pathway and/or *CD79B* and *TNFAIP3/A20* [14, 15]. This profile was recently classified as a specific type of DLBCL with mutations of the *MYD88*, *CD79B* and *PIM1* genes (MCD- or C5-enriched in cases with a NGC profile) [26, 27]. PCFCL, LC was characterized by a different mutational profile involving *TNFRSF14*, *B2M*, *CREBBP* and *EP300*, as recently described by two other groups in a common PCFCL subtype [12, 23]. In contrast to systemic follicular lymphoma and the GC subtype of DLBCL, no recurrent *EZH2* somatic mutation was found in our PCFCL, LC cases, in accordance with its rarity in PCFCL [12, 23, 28]. The wild-type profile was found in 19% of PCFCL, LC cases (n = 4 of 21), suggesting that our amplicon-based analysis may have missed them, unlike studies using massive exome and transcriptome parallel sequencing. The rate of mutations in our PCFCL cases was similar [26, 27]. However, mutations in chromatin-modifying genes identified in systemic follicular lymphoma are rarely detected in common PCFCL [12, 23]. Other methods such as single nucleotide polymorphism array or exome sequencing would determine if copy number variations permit to individualize PCFCL, LC from PCLBCL, LT. A new RT-MLPA assay involving NGS transcriptomic analysis of 137 markers and class prediction by machine learning was developed to classify most B-cell lymphoma subtypes; this commercially available technique may be able to reproduce our data [29].

Because retrospective studies may be biased depending on the inclusion criteria, we performed an integrative PCA based on limb involvement, immunophenotype, RT-MLPA profile, and mutational status, which categorized all cases into PCFCL, LC or PCLBCL, LT. Such integrative analysis could also be used by other groups to analyze the relevance of a cell-of-origin classification of PCLBCL, avoid the limitation of a single criterion or technique, and understand the discordance in results among countries.

Our findings indicate that cell-of-origin profiling of PCFCL, LC as GC, and PCLBCL, LT as non-GC, will be useful for dermatopathologists and hematopathologists, and is compatible with the use of cell of origin and the primary site for lymphoma classification [1, 30].

In the case of difficult-to-interpret skin FFPE samples, the RT-MLPA technique enables robust and cost-effective analysis of fixed specimens. Our data also suggest that a PCLBCL, NOS diagnosis should be avoided because RT-MLPA and/or mutational analysis may allow for a definitive diagnosis in conjunction with a simple non-hierarchical integrative analysis. More sophisticated tools based on RNA sequencing and artificial intelligence underscored the value of cell-of-origin profiling of DLBCL for patient stratification [31]. Such studies may be useful to predict the systemic spread of PCFCL, LC, similar to common PCFCL [23], as well as resistance to immunochemotherapy, as in patients with PCLBCL, LT [32].

## Supporting information

S1 Fig Mutational status determined by lymphopanel analysis of 55 primary cutaneous large B-cell lymphomas according to their histopathological diagnosis. PCLBCL, LT: primary cutaneous large B-cell lymphoma, leg type; PCFCL, LC: primary cutaneous follicle centre lymphoma, large cell; PCLBCL, NOS: primary cutaneous large B-cell lymphomas, not otherwise specified; GC: germinal centre; NGC: non germinal centre; RT-MLPA: reverse-transcriptase multiplex ligation analysis.
(TIF)

S1 Table Immunohistochemical profile and clinical features in 55 cases of primary cutaneous large B-cell lymphoma in the different diagnostic categories. PCLBCL, LT: primary cutaneous large B-cell lymphoma, leg type; PCFCL, LC: primary cutaneous follicle centre lymphoma, large cell; PCLBCL, NOS: primary cutaneous large B-cell lymphomas, not otherwise specified; FDCN: follicular dendritic cells network; ND: not done. M: male; F: female; RT: radiotherapy; S: surgery; RCT: rituximab-polychemotherapy; R: rituximab; No: no treatment; CR: complete remission; AWD: alive with disease; D+: dead from disease; D-: dead from other cause than disease.
(DOCX)

S2 Table Determination of cell of origin status (Germinal centre GC or Non-GC) according to the Hans’ algorithm, RT-MLPA assay and mutational pattern of 55 primary cutaneous large B-cell lymphomas. PCFCL, LC: primary cutaneous follicle centre lymphoma, large cell; PCLBCL, LT: primary cutaneous large B-cell lymphoma, leg type; PCLBCL, NOS: primary cutaneous large B-cell lymphoma, not otherwise specified; GC: germinal center. NC: non-contributive RT-MLPA assay. Mutational profile of PCFCL, LC is defined by *TNFRSF14* mutations; of PCLBCL, LT is defined by *MYD88* mutations or co-occurrence of *CD79B* and *PIM1* mutations. Nonspecific mutational profile defines profile without previously listed mutations. Molecular classification was based on RT-MLPA and mutational pattern concordant results or RT-MLPA profile only when no specific mutation was detected.
(DOCX)

## Abbreviations

DLBCL: Diffuse large B-cell lymphoma
EBV: Epstein-Barr virus
FFPE: Formalin-Fixed, Paraffin-Embedded
GC: Germinal centre
IHC: immunohistochemistry
NGS: Next Generation Sequencing
NF-κB: Nuclear factor-kappa B
OS: Overall survival
PCFCL: Primary cutaneous follicle centre lymphoma
PCFCL, LC: Primary cutaneous follicle centre lymphoma, large cell
PCLBCL: Primary cutaneous large B-cell lymphoma
PCLBCL, LT: Primary cutaneous large B-cell lymphoma, leg type
PCLBCL, NOS: Primary cutaneous large B-cell lymphoma, not otherwise specified
PCNSL: Primary Central Nervous System Lymphoma
RT-MLPA: Reverse transcriptase-multiplex ligation profiling assay
SS: Specific Survival
