# Supplementary figures and images for: Integrative diagnosis of primary cutaneous large B-cell lymphomas supports the relevance of cell of origin profiling
Source: PLoS One. 2022 Apr 22;17(4):e0266978. doi: 10.1371/journal.pone.0266978 (PMC9032422; doi:10.1371/journal.pone.0266978)

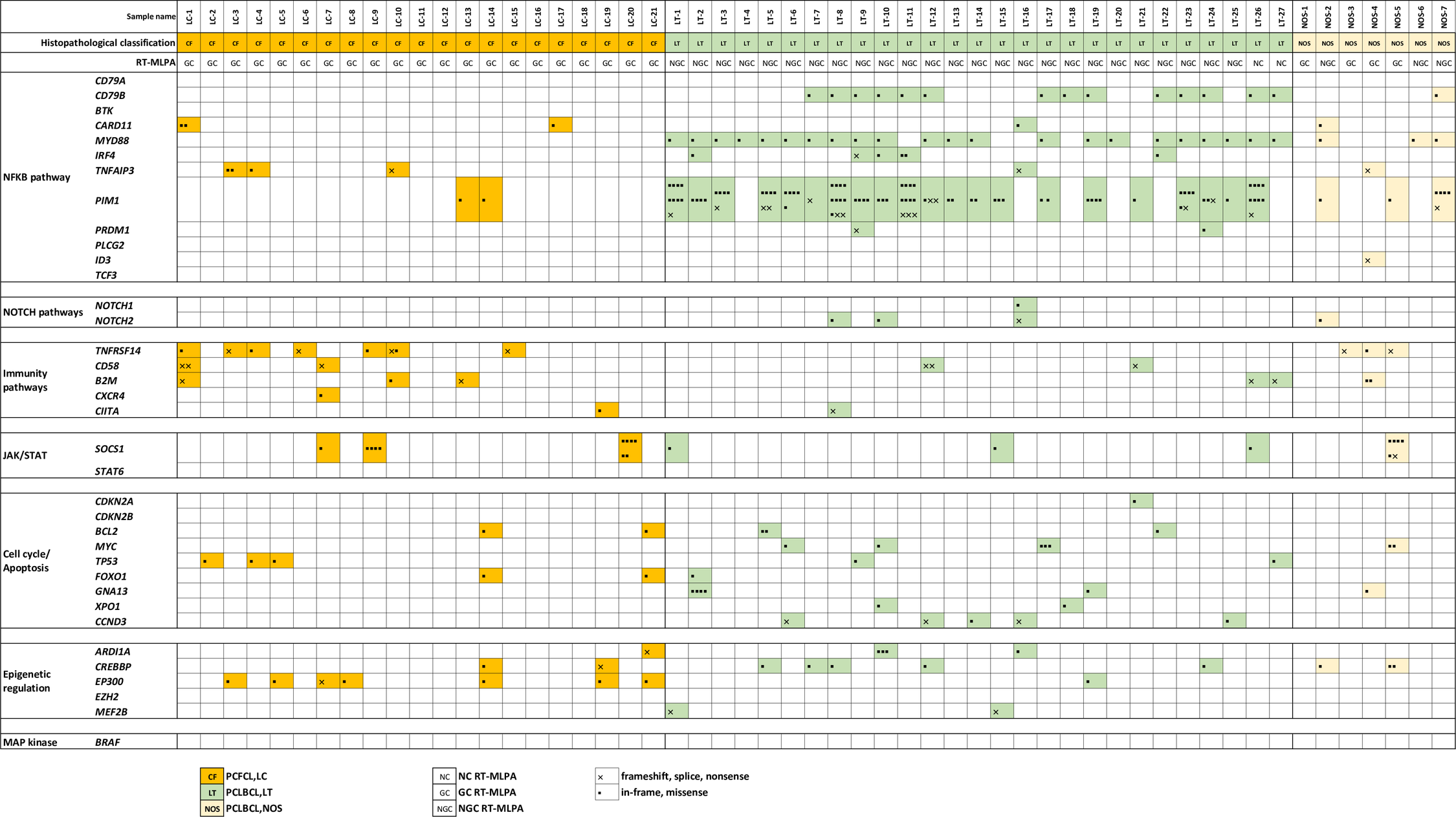

Supplement: S1 Fig — PCLBCL, LT: primary cutaneous large B-cell lymphoma, leg type; PCFCL, LC: primary cutaneous follicle centre lymphoma, large cell; PCLBCL, NOS: primary cutaneous large B-cell lymphomas, not otherwise specified; GC: germinal centre; NGC: non germinal centre; RT-MLPA: reverse-transcriptase multiplex ligation analysis. (TIF) [file pone.0266978.s001.tif]
