# Supplementary material for: Integrative diagnosis of primary cutaneous large B-cell lymphomas supports the relevance of cell of origin profiling
Source: PLoS One. 2022 Apr 22;17(4):e0266978. doi: 10.1371/journal.pone.0266978 (PMC9032422; doi:10.1371/journal.pone.0266978)
Supplement: S1 Table — PCLBCL, LT: primary cutaneous large B-cell lymphoma, leg type; PCFCL, LC: primary cutaneous follicle centre lymphoma, large cell; PCLBCL, NOS: primary cutaneous large B-cell lymphomas, not otherwise specified; FDCN: follicular dendritic cells network; ND: not done. M: male; F: female; RT: radiotherapy; S: surgery; RCT: rituximab-polychemotherapy; R: rituximab; No: no treatment; CR: complete remission; AWD: alive with disease; D+: dead from disease; D-: dead from other cause than disease. (DOCX) [file pone.0266978.s002.docx]

| **Case** | **WHO-EORTC Criteria** | **Age at diagnosis (years)** | **Gender** | **Location** | **T (TNM)** | **CD10** | **BCL6** | **MUM1** | **BCL2** | **IgM** | **FDCN**  **CD21+** | **Cutaneous relapse** | **Extra-cutaneous spreading, location** | **Initial treatment** | **Status at endpoint** | **Overall survival (months)** | **Progression free survival (months)** |
| --- | --- | --- | --- | --- | --- | --- | --- | --- | --- | --- | --- | --- | --- | --- | --- | --- | --- |
| LC-1 | PCFCL, LC | 60 | M | trunk | 1 | + | + | - | - | - | - | - | - | RT | CR | 102 | 102 |
| LC-2 | PCFCL, LC | 44 | M | head | 1 | - | + | - | - | - | - | + | - | S | CR | 33 | 3 |
| LC-3 | PCFCL, LC | 67 | F | head | 1 | + | + | - | - | - | + | - | - | RT | CR | 45 | 45 |
| LC-4 | PCFCL, LC | 41 | M | head | 2 | - | + | - | - | - | - | - | - | RCT | CR | 287 | 287 |
| LC-5 | PCFCL, LC | 70 | M | trunk | 3 | - | + | - | - | - | - | - | - | R | CR | 126 | 126 |
| LC-6 | PCFCL, LC | 83 | F | head | 2 | + | + | - | - | - | + | - | - | R | CR | 85 | 85 |
| LC-7 | PCFCL, LC | 63 | F | trunk | 1 | + | + | - | - | + | - | - | - | RT | CR | 32 | 32 |
| LC-8 | PCFCL, LC | 48 | M | head | 2 | - | + | - | - | - | + | + | +, bone | No | AWD | 52 | 41 |
| LC-9 | PCFCL, LC | 61 | M | trunk | 3 | + | + | - | + | - | - | - | - | RCT | CR | 40 | 40 |
| LC-10 | PCFCL, LC | 79 | F | head | 2 | + | + | - | - | - | + | + | - | RT | CR | 22 | 15 |
| LC-11 | PCFCL, LC | 69 | M | trunk | 2 | + | + | - | + | - | + | - | - | RT | CR | 39 | 39 |
| LC-12 | PCFCL, LC | 54 | M | trunk | 2 | - | + | - | + | - | - | + | - | R | CR | 30 | 7 |
| LC-13 | PCFCL, LC | 59 | M | trunk | 2 | - | + | - | - | - | + | - | +, regional lymph node | No | CR | 41 | 5 |
| LC-14 | PCFCL, LC | 45 | F | head | 2 | + | + | - | + | + | + | - | - | R | CR | 25 | 25 |
| LC-15 | PCFCL, LC | 64 | F | head | 1 | - | + | - | - | - | + | + | - | S | CR | 29 | 7 |
| LC-16 | PCFCL, LC | 48 | M | trunk | 2 | - | + | - | - | - | - | + | - | RT | AWD | 277 | 158 |
| LC-17 | PCFCL, LC | 65 | M | trunk | 1 | + | + | - | - | + | + | - | - | RT | CR | 13 | 13 |
| LC-18 | PCFCL, LC | 56 | M | upper limbs | 1 | + | + | - | - | - | - | + | +, regional lymph node | RT | CR | 49 | 10 |
| LC-19 | PCFCL, LC | 75 | M | trunk | 1 | - | + | - | - | - | - | + | - | No | CR | 27 | 16 |
| LC-20 | PCFCL, LC | 61 | M | head | 1 | + | + | - | + | + | + | - | - | RT | CR | 14 | 14 |
| LC-21 | PCFCL, LC | 85 | M | neck | 1 | + | + | - | + | - | - | - | - | R | CR | 14 | 14 |
| LT-1 | PCLBCL, LT | 82 | M | upper limb | 2 | - | + | + | + | - | - | + | +, deep lymph node | RT | D+ | 22 | 13 |
| LT-2 | PCLBCL, LT | 88 | F | lower limb | 2 | - | + | + | + | + | - | - | - | RCT | CR | 34 | 34 |
| LT-3 | PCLBCL, LT | 83 | F | upper limb | 2 | - | + | + | + | - | - | - | +, spleen, liver | RT | D+ | 16 | 15 |
| LT-4 | PCLBCL, LT | 95 | F | lower limb | 1 | - | + | + | + | - | - | - | - | RT | D- | 14 | 14 |
| LT-5 | PCLBCL, LT | 88 | F | lower limb | 3 | - | + | + | + | + | - | - | - | RCT | D- | 2 | 2 |
| LT-6 | PCLBCL, LT | 82 | M | lower limb | 2 | - | - | + | + | - | - | + | +, brain | RT | D+ | 20 | 8 |
| LT-7 | PCLBCL, LT | 87 | F | lower limb | 2 | - | - | + | + | ND | - | - | - | RCT | D+ | 11 | 11 |
| LT-8 | PCLBCL, LT | 90 | M | lower limb | 2 | - | + | + | + | + | - | - | +, nasal cavity | RCT | CR | 44 | 13 |
| LT-9 | PCLBCL, LT | 75 | F | lower limb | 3 | - | - | + | + | ND | - | - | +, regional and deep lymph node | RCT | D+ | 19 | 6 |
| LT-10 | PCLBCL, LT | 78 | F | lower limb | 2 | - | + | + | + | - | - | - | +, paravertebral mass | RCT | CR | 42 | 36 |
| LT-11 | PCLBCL, LT | 75 | M | upper limb | 3 | - | + | + | + | ND | - | - | - | RCT | D+ | 20 | 20 |
| **Case** | **WHO-EORTC Criteria** | **Age at diagnosis (years)** | **Gender** | **Location** | **T (TNM)** | **CD10** | **BCL6** | **MUM1** | **BCL2** | **IgM** | **FDCN**  **CD21+** | **Cutaneous relapse** | **Extra-cutaneous spreading, location** | **Initial treatment** | **Status at endpoint** | **Overall survival (months)** | **Progression free survival (months)** |
| LT-12 | PCLBCL, LT | 81 | F | trunk | 3 | - | + | + | + | + | - | + | +, deep lymph node | RCT | D+ | 17 | 9 |
| LT-13 | PCLBCL, LT | 89 | F | lower limb | 2 | - | + | + | + | - | - | - | - | RCT | D- | 26 | 26 |
| LT-14 | PCLBCL, LT | 73 | F | lower limb | 2 | - | + | + | + | - | - | - | - | No | D- | 4 | 4 |
| LT-15 | PCLBCL, LT | 61 | M | lower limb | 2 | - | + | + | + | - | - | + | - | RCT | AWD | 122 | 122 |
| LT-16 | PCLBCL, LT | 90 | M | lower limb | 2 | - | + | + | + | + | - | + | +, regional lymph node, bone | RCT | D+ | 19 | 10 |
| LT-17 | PCLBCL, LT | 83 | M | lower limb | 2 | - | - | + | + | ND | - | - | - | RCT | D+ | 12 | 12 |
| LT-18 | PCLBCL, LT | 62 | F | lower limb | 2 | - | - | + | + | ND | - | + | +, brain | RCT | D+ | 19 | 8 |
| LT-19 | PCLBCL, LT | 94 | M | lower limb | 2 | - | + | + | + | + | - | - | - | RCT | CR | 15 | 15 |
| LT-20 | PCLBCL, LT | 88 | F | lower limb | 2 | - | + | + | + | + | - | - | - | RCT | CR | 9 | 9 |
| LT-21 | PCLBCL, LT | 79 | M | lower limb | 1 | - | + | + | + | - | - | + | - | RCT | CR | 59 | 16 |
| LT-22 | PCLBCL, LT | 63 | F | lower limb | 2 | - | - | + | + | + | - | - | - | RCT | CR | 98 | 98 |
| LT-23 | PCLBCL, LT | 90 | F | lower limb | 3 | - | + | + | + | ND | - | - | +, deep lymph node | RCT | CR | 12 | 2 |
| LT-24 | PCLBCL, LT | 83 | F | trunk | 2 | - | - | + | + | ND | - | - | - | RCT | D- | 87 | 87 |
| LT-25 | PCLBCL, LT | 68 | F | lower limb | 1 | - | + | + | + | - | - | - | - | RCT | CR | 45 | 45 |
| LT-26 | PCLBCL, LT | 89 | M | lower limb | 2 | - | + | + | + | + | - | + | - | RCT | D+ | 50 | 22 |
| LT-27 | PCLBCL, LT | 80 | F | lower limb | 1 | - | + | + | + | - | - | - | - | RT | CR | 8 | 8 |
| NOS-1 | PCLBCL, NOS | 42 | M | trunk | 1 | - | + | + | + | **-** | - | - | - | RT | CR | 65 | 65 |
| NOS-2 | PCLBCL, NOS | 88 | F | lower limb | 1 | + | + | + | + | + | - | + | - | S | CR | 62 | 25 |
| NOS-3 | PCLBCL, NOS | 78 | F | lower limb | 1 | - | - | + | + | ND | - | - | - | RCT | CR | 29 | 29 |
| NOS-4 | PCLBCL, NOS | 74 | F | neck | 2 | - | + | - | - | - | - | - | - | RCT | CR | 24 | 24 |
| NOS-5 | PCLBCL, NOS | 83 | F | trunk | 1 | - | + | - | + | - | - | + | - | RCT | D+ | 32 | 21 |
| NOS-6 | PCLBCL, NOS | 72 | F | head | 2 | - | + | + | + | + | + | - | - | RT | CR | 14 | 14 |
| NOS-7 | PCLBCL, NOS | 81 | M | lower limb | 1 | - | - | + | + | - | - | - | - | RCT | CR | 59 | 59 |
