# Supplementary material for: Integrative diagnosis of primary cutaneous large B-cell lymphomas supports the relevance of cell of origin profiling
Source: PLoS One. 2022 Apr 22;17(4):e0266978. doi: 10.1371/journal.pone.0266978 (PMC9032422; doi:10.1371/journal.pone.0266978)
Supplement: S2 Table — PCFCL, LC: primary cutaneous follicle centre lymphoma, large cell; PCLBCL, LT: primary cutaneous large B-cell lymphoma, leg type; PCLBCL, NOS: primary cutaneous large B-cell lymphoma, not otherwise specified; GC: germinal center. NC: non-contributive RT-MLPA assay. Mutational profile of PCFCL, LC is defined by TNFRSF14 mutations; of PCLBCL, LT is defined by MYD88 mutations or co-occurrence of CD79B and PIM1 mutations. Nonspecific mutational profile defines profile without previously listed mutations. Molecular classification was based on RT-MLPA and mutational pattern concordant results or RT-MLPA profile only when no specific mutation was detected. (DOCX) [file pone.0266978.s003.docx]

|  | **Clinicopathological and Immunohistological profiling** | | **Molecular profiling** | | |
| --- | --- | --- | --- | --- | --- |
| **Case** | **WHO-EORTC criteria prior to molecular analyses** | **HANS** | **RT-MLPA** | **Mutational pattern** | **Molecular classification** |
| LC-1 | PCFCL, LC | GC | GC | PCFCL, LC | PCFCL, LC |
| LC-2 | PCFCL, LC | GC | GC | Non specific | PCFCL, LC |
| LC-3 | PCFCL, LC | GC | GC | PCFCL, LC | PCFCL, LC |
| LC-4 | PCFCL, LC | GC | GC | PCFCL, LC | PCFCL, LC |
| LC-5 | PCFCL, LC | GC | GC | Non specific | PCFCL, LC |
| LC-6 | PCFCL, LC | GC | GC | PCFCL, LC | PCFCL, LC |
| LC-7 | PCFCL, LC | GC | GC | Non specific | PCFCL, LC |
| LC-8 | PCFCL, LC | GC | GC | Non specific | PCFCL, LC |
| LC-9 | PCFCL, LC | GC | GC | PCFCL, LC | PCFCL, LC |
| LC-10 | PCFCL, LC | GC | GC | PCFCL, LC | PCFCL, LC |
| LC-11 | PCFCL, LC | GC | GC | No mutation | PCFCL, LC |
| LC-12 | PCFCL, LC | GC | GC | No mutation | PCFCL, LC |
| LC-13 | PCFCL, LC | GC | GC | Nonspecific | PCFCL, LC |
| LC-14 | PCFCL, LC | GC | GC | Nonspecific | PCFCL, LC |
| LC-15 | PCFCL, LC | GC | GC | PCFCL, LC | PCFCL, LC |
| LC-16 | PCFCL, LC | GC | GC | No mutation | PCFCL, LC |
| LC-17 | PCFCL, LC | GC | GC | Non specific | PCFCL, LC |
| LC-18 | PCFCL, LC | GC | GC | No mutation | PCFCL, LC |
| LC-19 | PCFCL, LC | GC | GC | Non specific | PCFCL, LC |
| LC-20 | PCFCL, LC | GC | GC | Non specific | PCFCL, LC |
| LC-21 | PCFCL, LC | GC | GC | Non specific | PCFCL, LC |
| LT-1 | PCLBCL, LT | Non-GC | Non-GC | PCLBCL, LT | PCLBCL, LT |
| LT-2 | PCLBCL, LT | Non-GC | Non-GC | PCLBCL, LT | PCLBCL, LT |
| LT-3 | PCLBCL, LT | Non-GC | Non-GC | PCLBCL, LT | PCLBCL, LT |
| LT-4 | PCLBCL, LT | Non-GC | Non-GC | PCLBCL, LT | PCLBCL, LT |
| LT-5 | PCLBCL, LT | Non-GC | Non-GC | PCLBCL, LT | PCLBCL, LT |
| LT-6 | PCLBCL, LT | Non-GC | Non-GC | PCLBCL, LT | PCLBCL, LT |
| LT-7 | PCLBCL, LT | Non-GC | Non-GC | PCLBCL, LT | PCLBCL, LT |
| LT-8 | PCLBCL, LT | Non-GC | Non-GC | PCLBCL, LT | PCLBCL, LT |
| LT-9 | PCLBCL, LT | Non-GC | Non-GC | PCLBCL, LT | PCLBCL, LT |
| LT-10 | PCLBCL, LT | Non-GC | Non-GC | PCLBCL, LT | PCLBCL, LT |
| LT-11 | PCLBCL, LT | Non-GC | Non-GC | PCLBCL, LT | PCLBCL, LT |
| LT-12 | PCLBCL, LT | Non-GC | Non-GC | PCLBCL, LT | PCLBCL, LT |
| LT-13 | PCLBCL, LT | Non-GC | Non-GC | PCLBCL, LT | PCLBCL, LT |
| LT-14 | PCLBCL, LT | Non-GC | Non-GC | PCLBCL, LT | PCLBCL, LT |
| LT-15 | PCLBCL, LT | Non-GC | Non-GC | Non specific | PCLBCL, LT |
| LT-16 | PCLBCL, LT | Non-GC | Non-GC | Non specific | PCLBCL, LT |
| LT-17 | PCLBCL, LT | Non-GC | Non-GC | PCLBCL, LT | PCLBCL, LT |
| LT-18 | PCLBCL, LT | Non-GC | Non-GC | Nonspecific | PCLBCL, LT |
| LT-19 | PCLBCL, LT | Non-GC | Non-GC | PCLBCL, LT | PCLBCL, LT |
| LT-20 | PCLBCL, LT | Non-GC | Non-GC | PCLBCL, LT | PCLBCL, LT |
| LT-21 | PCLBCL, LT | Non-GC | Non-GC | Nonspecific | PCLBCL, LT |
| LT-22 | PCLBCL, LT | Non-GC | Non-GC | PCLBCL-LT | PCLBCL, LT |
| LT-23 | PCLBCL, LT | Non-GC | Non-GC | PCLBCL, LT | PCLBCL, LT |
| LT-24 | PCLBCL, LT | Non-GC | Non-GC | PCLBCL, LT | PCLBCL, LT |
| LT-25 | PCLBCL, LT | Non-GC | Non-GC | PCLBCL, LT | PCLBCL, LT |
| LT-26 | PCLBCL, LT | Non-GC | NC | PCLBCL, LT | PCLBCL, LT |
| LT-27 | PCLBCL, LT | Non-GC | NC | PCLBCL, LT | PCLBCL, LT |
| NOS-1 | PCLBCL, NOS | Non-GC | GC | No mutation | PCFCL, LC |
| NOS-2 | PCLBCL, NOS | GC | Non-GC | PCLBCL, LT | PCLBCL, LT |
| NOS-3 | PCLBCL, NOS | Non-GC | GC | PCFCL, LC | PCFCL, LC |
| NOS-4 | PCLBCL, NOS | GC | GC | PCFCL, LC | PCFCL, LC |
| NOS-5 | PCLBCL, NOS | GC | GC | PCFCL, LC | PCFCL, LC |
| NOS-6 | PCLBCL, NOS | Non-GC | Non-GC | PCLBCL, LT | PCLBCL, LT |
| NOS-7 | PCLBCL, NOS | Non-GC | Non-GC | PCLBCL, LT | PCLBCL, LT |
